# Modulatory Effect of Probiotics on Proinflammatory Cytokine Levels in Acrylamide-Treated Rats

**DOI:** 10.1155/2021/2268770

**Published:** 2021-07-20

**Authors:** Seyed Mohammad Seifati, Erfan Zaker, Farzaneh Fesahat, Fateme Zare, Seyedhossein Hekmatimoghaddam

**Affiliations:** ^1^Student Research Committee, Shahid Sadoughi University of Medical Sciences, Yazd, Iran; ^2^Reproductive Immunology Research Center, Shahid Sadoughi University of Medical Sciences, Yazd, Iran; ^3^Department of Medical Genetics, School of Medicine, Shahid Sadoughi University of Medical Sciences, Yazd, Iran; ^4^Department of Advanced Medical Sciences and Technologies, School of Paramedicine, Shahid Sadoughi University of Medical Sciences, Yazd, Iran

## Abstract

The aims of this study are to investigate the effect of acrylamide on the level of proinflammatory cytokines in the blood of acrylamide-treated rats and to find the modulatory impact of probiotics on those cytokines. Thirty-two rats were divided into four groups: rats which received 20 mg acrylamide, acrylamide with 20 mg probiotics, acrylamide with 200 mg probiotics, and standard water and food (groups 1–4, respectively). The serum levels of cytokines were measured on days 0, 15, and 30. Group 1 showed an increased serum level of IL-1*β*, IL-6, and TNF-*α* after 15 days, and they decreased in day 30. Serum IL-6 level was significantly decreased on days 15 and 30 in rats in group 2 compared to the controls. TNF-*α* and IL-1*β* levels were not statistically different after treated with probiotics. The exposure of rats to acrylamide led to increased systemic inflammation as evidenced by higher levels of proinflammatory cytokines, and probiotics can modulate this inflammation.

## 1. Introduction

Acrylamide is a chemical monomer that is widely used as an additive in the textile, paper, and cosmetics industries. It is formed through the processing of high-carbohydrate-rich foods. The main route for the formation of acrylamide in heated foods is the Maillard reaction between the free amino group of the asparagine and a carbonyl source, such as sugars [1–4]. The carcinogenic effects of this substance are proven in previous studies [5, 6]. Acrylamide breaks down in the body and produces glycidamide. Glycidamide attacks DNA and causes mutations in genes [7, 8]. According to Pan et al., exposure to acrylamide causes oxidative stress marked by a significant increase in reactive oxygen species (ROS), malondialdehyde (MDA), and glutathione (GSH) consumption. Acrylamide activates signaling pathways of nuclear transcription factor 2-related to E2 (Nrf2) and nuclear factor-kB (NF-*κ*B). The mitogen-activated protein kinase (MAPK) pathway is also activated before activation of the Nrf2 and NF-*κ*B pathways. The inflammatory response is based on the level of proinflammatory cytokines, such as tumor necrosis factor-*α* (TNF-*α*) and interleukin-6 (IL-6) [9]. One study investigated oxidative stress, inflammation, and histopathological changes in hepatotoxicity due to concomitant use of acrylamide and ellagic acid (a natural phenolic constituent in some fruits and nuts posing antimutagenic, antiviral, anticancer, antitumor, and antioxidant properties) in Wistar rats. It was observed that administration of acrylamide increased levels of alanine transaminase (ALT), aspartate transaminase (AST), alkaline phosphatase (ALP), nitric oxide (NO), protein carbonyl (PC), MDA, TNF-*α*, and IL-1*β*. Also, acrylamide administration significantly decreased hepatic GSH level, superoxide dismutase (SOD), glutathione peroxidase (GPx), and catalase (CAT) activity when compared to the control group. It was shown that concomitant use of ellagic acid (30 mg/kg) with acrylamide significantly decreases levels of ALT, AST, ALP, NO, PC, TNF-*α* and IL-1*β* levels and also GSH contents so that they approached the control group levels. Meanwhile, GPx activity increased, but SOD and CAT activity did not significantly increase [10].

The term probiotic is defined by a committee of experts as microorganisms that have healthy effects on the host given that they enter the intestine in sufficient numbers [11, 12]. The positive effects of probiotics on humans include increasing tolerance and digestion of lactose and food, lowering intestinal pH, improving intestinal function, lowering cholesterol, ammonia, and other toxic compounds, producing B vitamins such as folic acid, repairing and restoring the normal intestinal flora after antibiotic treatment, treating and preventing acute diarrhea, increasing resistance to infections, antimutagenic and anticancer properties, and strengthening the immune system [13, 14]. Taking probiotics alone or consuming foods enriched with probiotics may reduce oxidative damage and free radicals [15]. According to a meta-analysis that investigated the effect of probiotics on inflammatory biomarkers, probiotic supplementation was significantly effective in reducing serum concentrations of C-reactive protein (CRP), TNF-*α*, IL-6, IL-12, and IL-4. Also, serum IL-10 concentrations increased significantly following the use of probiotic supplements [16]. Another meta-analysis studied the effects of probiotics on nonalcoholic fatty liver disease and found that probiotic therapy significantly reduced ALT, AST, and TNF-*α* [17]. Administration of probiotics can reduce A*β*1-40 plaques in rats with Alzheimer's disease [18]. Moreover, the expression of genes involved in the production of proinflammatory cytokines, including monocyte chemoattractant protein-1 (MCP-1), TNF-*α*, IL-1*β*, and IL-6 downregulated in the epididymis and hepatic adipose tissues of high-fat diet-induced obese mice treated with *Lactiplantibacillus plantarum* [19]. However, the anti-inflammatory effect of probiotics in acrylamide-treated rats has not been well studied.

This study aimed to investigate the effect of acrylamide on the level of proinflammatory cytokines in the blood of acrylamide-treated rats and to find the modulatory impact of probiotics on those cytokines.

## 2. Materials and Methods

### 2.1. Animals and Experimental Design

**Figure 1:**
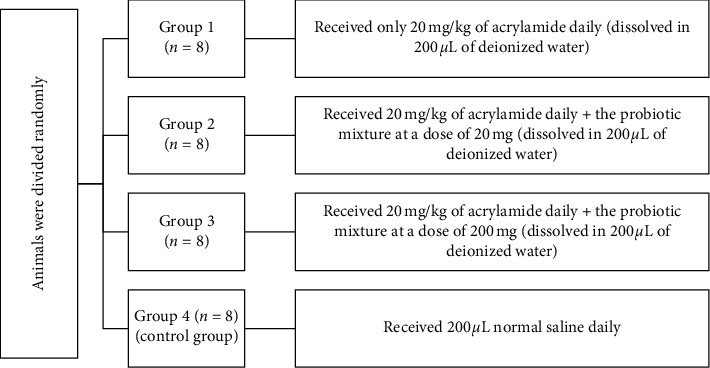
Schematic diagram of the categorization of the study groups.

Thirty-two male Wistar rats with an average weight of 300 ± 50 gr were purchased from the animal house of the Yazd Reproductive Sciences Institute, Shahid Sadoughi University of Medical Sciences in Yazd, Iran. The study was approved by the ethics committee of Shahid Sadoughi University of Medical Sciences in Yazd, Iran (ethics code no.: IR.SSU.REC.1400.023). All rats were kept in 12/12 light/dark cycles, at 22°C, 55% humidity, and without restriction on water and food for 30 days. The random allocation of rats and the administered doses of acrylamide and probiotics, all through gavage, are shown in Figure 1.

### 2.2. Probiotic Strains

The bacteria used in this study included probiotic bacteria in one-gram sachets (Kidilact, Zist Takhmir Co., Tehran, Iran), which are commercially used for children over 2 years of age and contain high amounts of 7 beneficial bacterial strains besides fructooligosaccharide (FOS). FOS promotes the growth and activity of probiotics. The bacterial count of this product is 10^9^ CFU, and the applied strains biotics include the following: *Lactobacillus casei, Lactobacillus acidophilus, Lactobacillus rhamnosus, Lactobacillus bulgaricus, Bifidobacterium infantis, Bifidobacterium breve,* and *Streptococcus thermophilus*.

### 2.3. Cytokine Concentration Assay

Blood samples were taken from rats' retrobulbar plexus on days 0, 15, and 30 by a capillary tube. The serum levels of IL-1*β*, IL-6, and TNF-*α* were measured using an enzyme-linked immunosorbent assay kit (Karmania Pars Gene, Kerman, Iran) and according to the kit instructions. The sensitivities of IL-1*β*, IL-6, and TNF-*α* were 8 pg/mL, 2 pg/mL, and 4 pg/mL, respectively.

### 2.4. Statistical Analysis

All concentrations were expressed as mean ± standard error of the mean (SEM). Data were tested for normality of distribution and equality of variances by the Shapiro–Wilk test using Statistical Software for the Social Sciences (SPSS, IBM, US), version 20. To compare the results across the study groups before and after each intervention, the paired sample *t*-test and the Mann–Whitney *U*-test were performed. The one-way ANOVA test followed by Tukey's post hoc analysis was used for comparing other variables. *P* < 0.05 was considered as a significant value.

## 3. Results

The serum concentrations of IL-1*β*, IL-6, and TNF-*α* (pg/mL) in all groups are represented in Tables 1–3, respectively. It was shown that acrylamide (20 mg/kg) increased the serum level of IL-1*β* gradually after 15 days and then gradually decreased until the end of the 30-day study period. Additionally, on the 30^th^ day of the intervention, the serum IL-1*β* levels were significantly higher in the first group compared to the control group (*P*=0.03).

Table 2 demonstrates that acrylamide (20 mg/kg) increased the serum level of IL-6 gradually after 15 days. However, at the end of the 30-day study period, it was slightly lower than the level at day 0. Serum IL-6 levels were significantly decreased on days 15 and 30 of the study by administration of acrylamide and probiotics (20 mg) compared with the control group (*P*=0.04, *P*=0.006, respectively).

Table 3 shows that acrylamide (20 mg/kg) increased the serum level of TNF-*α* until day 15 with the decreased level at the end of the 30-day study period, even slightly less than its level on the first day. TNF-*α* levels were not different statistically across studied groups throughout the study.

## 4. Discussion

Cytokines are small molecules that have important roles in cell signaling. Cytokines are now recognized as major regulators of growth, differentiation, and immune cell function in infectious and chronic inflammatory conditions. Proinflammatory cytokines, such as IL-1*β*, IL-6, and TNF-*α* regulate the onset of inflammation reactions [20]. IL-1*β* affects almost every cell type and is often in coordination with TNF-*α* [21, 22]. IL-1*β*, IL-6, and TNF-*α* are all secreted by macrophage and endothelial cells as part of the innate immune system and proinflammatory cytokines. In general, in the first stage of inflammation, these cytokines are responsible for causing fever by their action on the hypothalamus, synthesis of acute-phase proteins, and activation of endothelial cells and neutrophils [23]. One of the currently hot topics about reducing the inflammatory effects is probiotics.

Probiotics are living microorganisms (bacteria and yeasts) that live in the human gastrointestinal tract and improve host health by fermenting undigested food. They have further beneficial roles such as antipathogenic, antiobesity, antidiabetic, anti-inflammatory, and anticancer properties. In addition, they can improve angiogenic activities in the brain and central nervous system [24, 25]. In the present study, we evaluated the protective effects of probiotics on proinflammatory cytokine levels in acrylamide-treated rats. Acrylamide is a hazardous chemical that is widely used in industrial practices, and also, this toxic substance can be found in baked, fried foods, and cigarette and tobacco which is responsible for desirable flavor and color in fried foods [26–28]. Neurotoxicity, reproductive toxicity, and genotoxicity are the most evident complications of acrylamide observed in animals and humans [29–31]. It is observed that, during the metabolism of acrylamide throughout the body, excessive levels of reactive oxygen species (ROS) are certainly produced. In addition, acrylamide intoxication may have an increasing effect on the production of proinflammatory cytokines such as TNF-*α* and IL-1*β* [32, 33]. Our results indicated that exposing rats to a fixed dose of acrylamide along with the probiotic mixture at a dose of 20 mg led to a significant decrease in the concentration of IL-1*β* after 30 days. The first group, who received 20 mg/kg acrylamide daily, had higher IL-1 *β* levels than the control group on day 30. So, it can be concluded that acrylamide induces the production of this proinflammatory cytokine. Similarly, a study on acrylamide-treated Swiss albino mice found that acrylamide increased the level of proinflammatory cytokines such as TNF-*α* and IL-1*β* [32]. Ghorbel et al., [15] found a significant increase in TNF-*α*, IL-1*β*, and IL-6 in the liver of rats treated with acrylamide compared with controls. In contrast, our results unexpectedly showed that the serum level of IL-6 in the group treated with acrylamide was significantly lower compared to the control group at all times. Furthermore, no significant change in serum TNF-*α* levels was observed in all groups, denoting that this cytokine would not be helpful in the evaluation of the impact of acrylamide on rats. However, May N. Bin-Jumah et al. reported a markedly increased TNF-*α* levels in their study [26].

## 5. Conclusion

The present study provided interesting new observations for the possibility of beneficial health effects by coadministration of probiotics for acrylamide-exposed rats. Our findings suggested that exposure of rats to acrylamide led to increased systemic inflammation as evidenced by higher levels of proinflammatory cytokines. Studies on humans regarding the possible positive impacts of probiotics in this field could complement our work.

## Figures and Tables

**Table 1:** Effect of treatment with the probiotic on the serum levels of IL-1*β* at different times and across all study groups.

Groups	IL-1*ß*_0_ (pg/mL)	IL-1*ß*_15_ (pg/mL)	IL-1*ß*_30_ (pg/mL)	*P* value
1 (acrylamide)	57.88 ± 5.88	70.39 ± 25.59	45.14 ± 6.52	0.51^a^, 0.275^b^, 0.51^c^
2 (acrylamide + LD)	60.04 ± 6.51	72.92 ± 12.53	26.06 ± 2.66	0.275^a^, 0.05^b^, 0.05^c^
3 (acrylamide + HD)	43.72 ± 4.56	37.50 ± 15.33	29.94 ± 3.65	0.513^a^, 0.05^b^, 0.82^c^
4 (control)	63.05 ± 7.17	70.42 ± 13.54	23.87 ± 4.01	0.82^a^, 0.05^b^, 0.05^c^
*P* value^*∗*^	0.94	0.46	**0.03**	—

Data are presented as mean ± SEM (standard error of the mean). LD: low dose of probiotics; HD: high dose of probiotics; bold *P* values show a significant difference (*P* < 0.05). ^a^Comparison between days 0 and 15 (*t*-test). ^b^Comparison between days 0 and 30 (*t*-test). ^c^Comparison between days 15 and 30 (*t*-test). ^*∗*^Comparison of 4 groups (one-way ANOVA).

**Table 2:** Effect of treatment with the probiotic on the serum levels of IL-6 at different times and across all study groups.

Group	IL-6_0_ (pg/mL)	IL-6_15_ (pg/mL)	IL-6_30_ (pg/mL)	*P* value
1 (acrylamide)	78.83 ± 11.85	111.3 ± 40.77	38.02 ± 6.65	0.51^a^
				0.05^b^
				0.27^c^

2 (acrylamide + LD)	81.03 ± 11.17	26.97 ± 4.23	26.17 ± 3.37	0.05^a^
				0.05^b^
				0.82^c^

3 (acrylamide + HD)	98.68 ± 7.38	51.18 ± 14.85	45.02 ± 4.61	0.05^a^
				0.05^b^
				0.51^c^

4 (control)	114.1 ± 5.91	130.1 ± 20.51	93.95 ± 17.82	0.51^a^
				0.05^b^
				0.05^c^

*P* value^*∗*^	0.08	**0.04**	**0.006**	—

Data are presented as mean ± SEM (standard error of mean). LD: low dose of probiotics; HD: high dose of probiotics; bold *P* values show a significant difference (*P* < 0.05). ^a^Comparison between days 0 and 15. ^b^Comparison between the days 0 and 30. ^c^Comparison between days 15 and 30. ^*∗*^Comparison of 4 groups.

**Table 3:** Effect of treatment with the probiotic on the serum levels of TNF-*α* at different times and across all study groups.

Group	TNF-*α*_0_ (pg/mL)	TNF-*α*_15_ (pg/mL)	TNF-*α*_30_ (pg/mL)	*P* value
1 (acrylamide)	96.95 ± 7.49	108.0 ± 23.78	94.92 ± 8.73	0.82^a^
				0.82^b^
				0.82^c^

2 (acrylamide + LD)	95.68 ± 3.63	82.34 ± 7.37	96.07 ± 22.95	0.12^a^
				0.51^b^
				0.82^c^

3 (acrylamide + HD)	114.0 ± 5.56	119.7 ± 32.10	73.27 ± 11.04	0.82^a^
				0.05^b^
				0.27^c^

4 (control)	103.3 ± 3.93	100.2 ± 19.63	68.40 ± 4.48	0.82^a^
				0.05^b^
				0.51^c^

*P* value^*∗*^	0.17	0.78	0.25	—

Data are presented as mean ± SEM (standard error of mean). LD: low dose of probiotics; HD: high dose of probiotics; bold *P* values show a significant difference (*P* < 0.05). ^a^Comparison between days 0 and 15. ^b^Comparison between days 0 and 30. ^c^Comparison between days 15 and 30. ^*∗*^Comparison of 4 groups.

## Data Availability

The final analyzed data used to support the findings of this study are included within the article. The raw data would be available from the corresponding author upon request.
